# Perceived Knowledge, Clinical Skills, Self-Confidence, and Barriers Among Primary Health Care Physicians in Performing Minor Surgical Procedures and Intramuscular Injections in Riyadh, Saudi Arabia

**DOI:** 10.7759/cureus.112247

**Published:** 2026-07-08

**Authors:** Faten Almazroa, Shrouq Almarzooqi, Reem Alrasheedi, Bandar Alshehry, Suha Algain

**Affiliations:** 1 Family Medicine, King Saud Medical City, Riyadh, SAU

**Keywords:** family medicine, general practitioner, intramuscular injections, minor surgical procedures (msp), primary healthcare, procedure training, riyadh, saudi arabia

## Abstract

Background

Minor surgical procedures (MSPs) and intramuscular (IM) injections are important components of family medicine for managing dermatological and musculoskeletal conditions in primary care. Performing these procedures in primary healthcare centers can improve patient comfort, reduce healthcare costs, and enhance continuity of care. However, the practice of MSPs and IM injections among family physicians in Riyadh, Saudi Arabia, remains limited, with barriers including insufficient training, inadequate facilities, and low self-confidence.

Methods

A cross-sectional study was conducted among family physicians working in Ministry of Health hospitals, primary healthcare, private sector, and other settings in Riyadh, Saudi Arabia. A convenience sampling approach was employed. Based on a total population of 872 physicians, the minimum required sample size was calculated as 267. A total of 269 completed responses were ultimately included in the analysis. Data were collected using a structured online questionnaire. The survey assessed demographics, perceived knowledge, skills, confidence, and perceived barriers related to performing MSPs and IM injections. Data were analyzed using descriptive and inferential statistics in IBM SPSS Statistics for Windows, version 31 (released 2025; IBM Corp., Armonk, New York, United States).

Results

A total of 269 primary healthcare physicians participated (mean age 33.24 ± 6.58 years) with nearly equal gender distribution. About 52.8% reported receiving formal training in minor surgical procedures. Overall perceived knowledge was moderate. However, the frequency of performing MSPs and IM injections was generally low, with many physicians reporting they rarely or never perform these procedures. Confidence in procedural skills, such as suturing superficial wounds and abscess drainage, ranged from low to moderate. The most commonly reported barriers were lack of training (n=184; 68.4%), lack of time (n=171; 63.6%), and lack of equipment (n=143; 53.2%). Workshops and hands-on training were the most frequently identified facilitators (n=209; 77.7%). Perceived knowledge showed a moderate positive correlation with confidence (ρ = 0.384, p < 0.01). Years of experience correlated moderately with perceived knowledge (ρ = 0.403, p < 0.001) and weakly with confidence (ρ = 0.165, p = 0.007).

Conclusion

Primary healthcare physicians demonstrated moderate perceived knowledge but limited confidence and practice in MSPs and IM injections. Structured hands-on training, improved workflow, adequate resources, and mentorship may help improve procedural capacity in primary care and reduce unnecessary specialist referrals.

## Introduction

Minor surgical procedures (MSP) and Intramuscular (IM) injections play a crucial role in family medicine because they are frequently encountered by primary care physicians. MSPs include any procedure that is performed relatively simply, usually under local anesthesia, has a short duration, involves a relatively accessible part of the body, and is rarely associated with intraoperative or postoperative complications [1]. These procedures are essential for managing both dermatological and IM injections, ranging from skin lesions like nevi, fibromas, lipomas, lacerations, ingrown nails, and abscesses [2,3]. IM injections are a commonly used parenteral route of medication administration in which drugs are delivered into skeletal muscle, allowing rapid systemic absorption due to the muscle’s rich vascular supply. It is widely used for the administration of vaccines, antibiotics, hormonal therapies, and other medications when oral or subcutaneous administration is not appropriate [4,5]

Performing MSPs and IM injections in the primary care setting has several advantages including: (a) anxiety reduction, since the procedure is performed by the patient's family physician and not a stranger, (b) greater convenience for the patient, due to proximity to and familiarity with the clinic, (c) financial savings, since procedures conducted in primary care clinics are less expensive than hospital procedures, and (d) shorter waiting times. Performing these procedures in the primary care setting may improve the physician-patient relationship and enable primary care physicians to increase the spectrum of their work, enhance their work satisfaction [6].

In Saudi Arabia, one study of primary healthcare (PHC) physicians conducted in Al-Qatif city explored physicians' perceptions, attitudes, and readiness with regard to MSPs. Most (86.9%) were interested in conducting MSPs in the PHC setting, but they were not confident in doing so. Reported barriers to performing MSPs were inadequate facilities (90.2%), staff shortages (55.8%), fear of complications (73.7%), medicolegal considerations (72.2%), lack of time (70.4%), and ease of referral to other specialties (57.3%). A significant number of physicians also reported lacking the necessary training (80%) and experience (65.4%) to perform MSPs [7]. Similarly, a cross-sectional study of Canadian family physicians found that a significant number do not usually perform the following types of MSPs: skin excision, shoulder or knee joint injections, and endometrial biopsy [8]. Instead, they refer patients to other specialties. The most common reasons given for doing this were lack of skills and lack of time. Likewise, a newly graduated physician may not feel confident enough to practice certain MSPs in the PHC setting because of insufficient exposure to those procedures during their medical school training or residency program. In contrast, a study conducted in Ireland in 2000 found that there was a significant decrease in the referral of MSPs to specialists following the completion of educational programs [9]. This finding is further supported by research from Lowy et al., which indicated that an increase in the number of procedures performed at the primary care level did not lead to a decline in the quality of care [10]. For those who prefer not to perform these procedures themselves, referring patients to another primary care provider who does perform them is an option that remains largely underutilized. 

These findings are further supported by evidence demonstrating the benefits of structured procedural training. Gmajnić et al. reported that participation in a practical surgical training course was associated with a significant increase in the number of minor surgical procedures performed by family physicians in their offices [11]. This suggests that structured hands-on training may enhance procedural competence and expand the scope of office-based surgical services provided in primary care.

This study aimed to evaluate the perceived knowledge, clinical skills, and self-confidence to perform MSPs and IM injections by family medicine physicians in Riyadh, Saudi Arabia, as, given the importance of practicing MSPs and IM injections in PHC, current research is required to clearly understand the gaps in the practice.

## Materials and methods

This was a cross-sectional study conducted in Riyadh, Saudi Arabia. The study was approved by the Institutional Review Board (IRB) of King Saud Medical City, Riyadh (approval number: H1RI-23-Jun25-01). Informed consent was obtained electronically from all participants prior to participation, and all responses were kept confidential and anonymous. Participation was voluntary, and participants were informed of their right to withdraw at any time without any consequences.

Study population

Eligible participants were physicians currently practicing PHC in Riyadh, including General Practitioners (GPs), Family Medicine residents, Family Medicine specialists, and Family Medicine consultants, working in Ministry of Health (MOH) government hospitals, MOH PHC centers, private healthcare facilities, or other PHC settings. Both Saudi and non-Saudi physicians, and both male and female physicians, were eligible to participate. Interns, medical students, consultants from specialties other than Family Medicine, and physicians not practicing in primary healthcare were excluded.

The total eligible population was determined as 872 from the MOH statistics. Using the Raosoft calculator (https://raosoftcalculator.com/), ensuring a margin of error of 5% and a confidence level of 95%, the sample size was calculated to be 267. However, a total of 269 responses were received and included in the final analysis. A convenience sampling approach was employed to ensure representation across different demographics, such as age, gender, nationality, qualification, experience in PHC, and place of work.

Data collection and study tool

Data were collected through an online survey (see Appendices) distributed via professional WhatsApp groups (Meta Platforms, Inc., Menlo Park, California, United States). The Questionnaire was divided into the following sections: (i) Demographics, (ii) Knowledge Assessment, (iii) Skills and Practice, (iv) Barriers, and (v) Suggestions for Improvement. A five-point Likert scale was used for the Knowledge Assessment (perceived knowledge) and Skills and Practice sections; while the former were scored from very poor to excellent, the latter were scored from never to always. Scores for each domain were calculated by adding the relevant items.

The perceived knowledge score included four items related to aseptic technique, indications for common MSPs, complications, and safe IM injection sites and techniques. The possible score ranged from 4 to 20. In the Skills and Practice section, the practice frequency score included two items on performing MSPs and IM injections, with a possible range of 2-10. The self-confidence score included three items on suturing superficial wounds, performing abscess drainage, and managing post-procedure complications, with a possible range of 3-15. The item on consistent use of sterilized equipment and standard precautions was analyzed separately as a single safety adherence item, scored from 1-5. In all the domains above, higher scores represented higher perceived knowledge, more frequent practice, or greater self-reported confidence. 

The questionnaire was reviewed by subject-matter experts before data collection. The review focused on item clarity, relevance, and coverage of the study objectives. The questionnaire underwent face and content validity assessment but did not undergo formal psychometric validation. 

Data analysis

Internal consistency was assessed using Cronbach’s alpha. The alpha values were 0.876 for perceived knowledge, 0.789 for practice frequency, and 0.717 for self-confidence. The alpha value for all 10 Likert-type items together was 0.861. A separate alpha value was not calculated for safety adherence because it was measured using one item only. The collected data and figures were analyzed and generated using IBM SPSS Statistics for Windows, version 31 (released 2025; IBM Corp., Armonk, New York, United States). 

Descriptive statistics were used to summarize participants' characteristics and responses; categorical variables were presented as frequencies and percentages, while continuous and ordinal variables were presented as means with standard deviations and as medians with interquartile ranges (IQR). Composite scores for perceived knowledge and confidence were computed by summing the relevant five-point Likert-scale items, and the internal consistency of each scale was assessed using Cronbach's alpha (perceived knowledge = 0.876; practice frequency = 0.789; self-confidence = 0.717; all 10 items = 0.861).

The distributional properties of the study variables were examined using the Kolmogorov-Smirnov and Shapiro-Wilk tests, supplemented by visual inspection of histograms and Q-Q plots. Formal testing indicated statistically significant deviations from normality for all knowledge, practice, and confidence items (Shapiro-Wilk, p < 0.001). Given this finding, together with the inherently ordinal nature of the Likert-derived measures, nonparametric methods were applied consistently throughout all inferential analyses. Because both composite scores significantly departed from normality (perceived knowledge: Shapiro-Wilk W = 0.981, p = 0.001; self-confidence: W = 0.968, p < 0.001), the nonparametric Mann-Whitney U test was used to compare independent groups in place of the parametric independent-samples t-test.

Specifically, the Mann-Whitney U test was used to compare composite perceived knowledge and confidence scores between independent groups (e.g., male vs. female physicians). Spearman's rank correlation coefficient (ρ) was used to examine bivariate associations among the composite scores and between these scores and years of experience in primary healthcare. Statistical significance was set at p < 0.05 (two-tailed).

## Results

Participant demographic and professional characteristics

**Figure 1:**
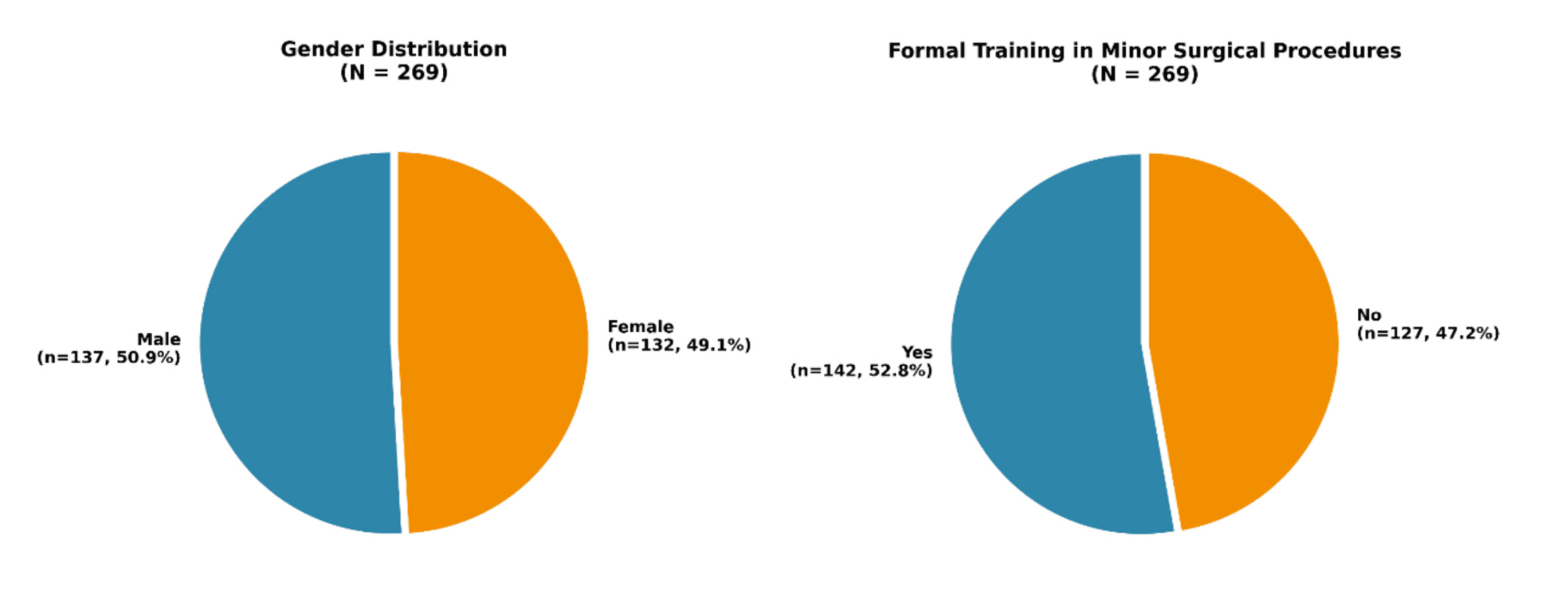
Participant demographic and professional characteristics

As shown in Figure 1, a total of 269 primary healthcare physicians were included in the analysis. The mean age of participants was 33.24 ± 6.58 years. The gender distribution was nearly equal, with 137 males (50.9%) and 132 females (49.1%). Most participants were Saudi nationals (87.4%), while 12.6% were non-Saudi.

Regarding workplace setting, the majority of physicians were employed in MOH PHCs (n = 148, 55.0%), followed by MOH hospitals (n = 102, 37.9%). A smaller proportion worked in the private sector (n = 15, 5.6%), and four physicians (1.5%) were affiliated with other settings, including mixed or non-MOH facilities such as private primary care clinics or university-affiliated centers.

In terms of professional preparation, 142 physicians (52.8%) reported having received formal training in minor surgical procedures, whereas 127 (47.2%) indicated that they had not received such training.

Perceived knowledge of MSPs and IM injections

Overall, knowledge levels among physicians were predominantly moderate across the assessed domains (Table 1). Most participants reported average or good knowledge of aseptic techniques, indications for procedures, and IM injection practices. However, a noticeable proportion of physicians reported poor or very poor knowledge, particularly in areas related to complications and injection techniques.

**Table 1:** Perceived knowledge of MSPs and IM injections among family medicine physicians (N = 269) NOTE: Percentages are calculated as n/269 × 100 and rounded to one decimal place; totals may not sum exactly to 100% due to rounding MSP: minor surgical procedure; IM: intramuscular

Item	Excellent, n (%)	Good, n (%)	Average, n (%)	Poor, n (%)	Very Poor, n (%)
Knowledge of aseptic techniques during MSP	18 (6.7)	62 (23.0)	119 (44.2)	52 (19.3)	18 (6.7)
Understanding indications for common MSP	28 (10.4)	75 (27.9)	119 (44.2)	38 (14.1)	9 (3.3)
Knowledge of MSP-related complications	30 (11.2)	83 (30.9)	88 (32.7)	56 (20.8)	12 (4.5)
Knowledge of safe IM injection sites and techniques	32 (11.9)	69 (25.7)	92 (34.2)	55 (20.4)	21 (7.8)

Practice frequency, confidence, and safety in performing MSPs and IM injections

**Figure 2:**
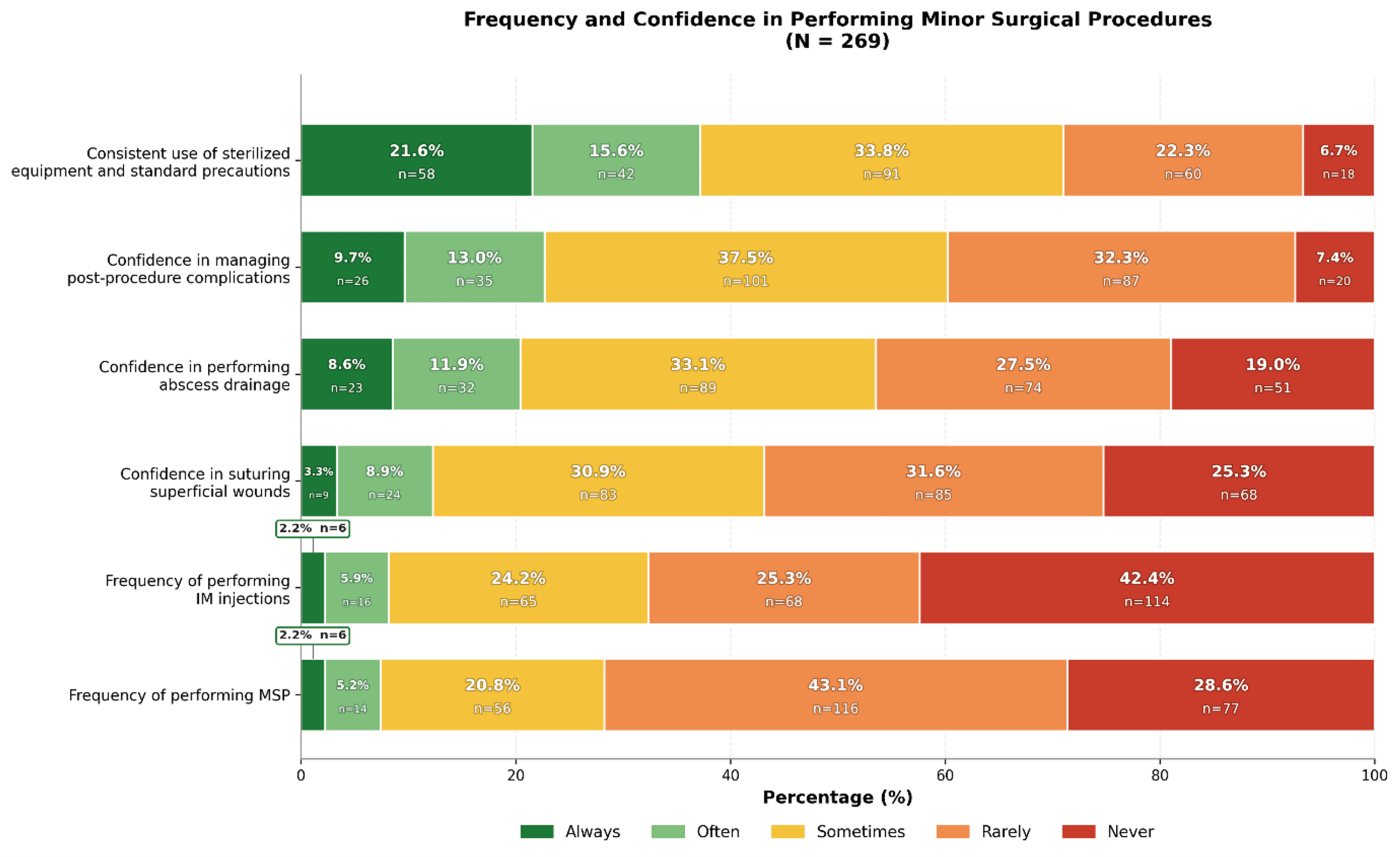
Practice frequency, confidence, and safety in performing MSPs and IM injections (N = 269) MSP: minor surgical procedures; IM: intramuscular

Table 2 and Figure 2 show that the frequency of performing MSPs and IM injections was generally low. A substantial proportion of physicians reported rarely or never performing these procedures. Confidence levels were moderate overall, with many participants reporting only occasional confidence in performing procedures such as suturing and abscess drainage. In contrast, adherence to safety practices, including the use of sterilized equipment and standard precautions, was relatively higher compared with other domains.

**Table 2:** Practice frequency, confidence, and safety in performing MSPs and IM injections (N = 269) NOTE: Percentages are calculated as n/269 × 100 and rounded to one decimal place; totals may not sum exactly to 100% due to rounding MSP: minor surgical procedure; IM: intramuscular

Item	Always n (%)	Often n (%)	Sometimes n (%)	Rarely n (%)	Never n (%)
Frequency of performing MSP	6 (2.2)	14 (5.2)	56 (20.8)	116 (43.1)	77 (28.6)
Frequency of performing IM injections	6 (2.2)	16 (5.9)	65 (24.2)	68 (25.3)	114 (42.4)
Confidence in suturing superficial wounds	9 (3.3)	24 (8.9)	83 (30.9)	85 (31.6)	68 (25.3)
Confidence in performing abscess drainage	23 (8.6)	32 (11.9)	89 (33.1)	74 (27.5)	51 (19.0)
Confidence in managing post-procedure complications	26 (9.7)	35 (13.0)	101 (37.5)	87 (32.3)	20 (7.4)
Consistent use of sterilized equipment and standard precautions	58 (21.6)	42 (15.6)	91 (33.8)	60 (22.3)	18 (6.7)

Barriers to performing MSPs and IM injections

The most commonly reported barriers were lack of training, lack of time, and lack of equipment or supplies, as presented in Table 3. Other reported barriers included institutional restrictions and fear of complications, while low patient demand was less frequently identified.

**Table 3:** Reported barriers to performing MSPs and IM injections (N = 269) MSP: minor surgical procedures; IM: intramuscular

Barrier	Frequency	Percentage
Lack of training	184	68.4
Lack of time	171	63.6
Lack of equipment / supplies	143	53.2
Institutional restrictions	82	30.5
Fear of complications	66	24.5
Low patient demand	60	22.3
Total participants	269	100.0

Factors that would facilitate the performance of MSPs and IM injections

Table 4 summarizes that participants identified several factors that could enhance their performance of procedures. The most frequently reported facilitators were workshops and hands-on training, followed by access to proper instruments and clear institutional guidelines. Supervisory support or mentorship was also commonly reported.

**Table 4:** Factors that would help physicians perform MSPs and IM injections more confidently and frequently (N = 269) MSP: minor surgical procedures; IM: intramuscular

Facilitating factor	Frequency	Percentage
Workshops / hands-on training	209	77.7
Access to proper instruments	156	58.0
Clear institutional guidelines	142	52.8
Supervisory support / mentorship	129	48.0
Total participants	269	100.0

Correlation between perceived knowledge and confidence scores

Table 5 shows that a statistically significant positive correlation was observed between perceived knowledge and confidence scores, indicating that higher knowledge levels were associated with higher confidence.

**Table 5:** Correlation between knowledge and confidence scores (N = 269) **correlation is statistically significant at the 0.01 level (two-tailed)

Variable	1	2
1. Perceived Knowledge score	1.000	0.384**
2. Confidence score	0.384**	1.000

Association of perceived knowledge and confidence scores with gender

As shown in Table 6, gender-based differences in composite perceived knowledge (SKT) and confidence (SST) scores were examined using the Mann-Whitney U test. The distributions of both scores were comparable between male and female physicians, and no statistically significant differences were observed.

**Table 6:** Comparison of composite perceived knowledge and confidence scores by gender (N = 269)

Outcome	Gender	Median (IQR)	Mean Rank	Mann–Whitney U	Z	p-value
Perceived knowledge score (SKT)	Male	12.00 (10.00– 16.00)	135.04	9048.00	– 0.009	0.993
	Female	12.00 (11.00– 15.00)	134.95			
Confidence score (SST)	Male	8.00 (6.00– 10.00)	138.97	9586.00	– 0.860	0.390
	Female	8.00 (6.00– 9.00)	130.88			

For the perceived knowledge score, male physicians (n = 137) had a median of 12.00 (IQR: 10.00-16.00) and a mean rank of 135.04, while female physicians (n = 132) had a median of 12.00 (IQR: 11.00-15.00) and a mean rank of 134.95 (U = 9048.00, Z = -0.009, p = 0.993). For the confidence score, male physicians had a median of 8.00 (IQR: 6.00-10.00) and a mean rank of 138.97, compared with a median of 8.00 (IQR: 6.00-9.00) and a mean rank of 130.88 for female physicians (U = 9586.00, Z = -0.860, p = 0.390).

Overall, these findings indicate that gender was not significantly associated with either perceived knowledge or self-reported confidence regarding the performance of minor surgical procedures and intramuscular injections.

Association of perceived knowledge and confidence scores with years of experience

As shown in Table 7, years of experience were positively associated with both knowledge and confidence scores. A moderate correlation was observed with perceived knowledge (ρ = 0.403, p < 0.001), while a weaker correlation was found with confidence (ρ = 0.165, p = 0.007)

**Table 7:** Correlation of years of experience with knowledge and confidence scores (N = 269) Spearman’s rank correlation was used. ** p < 0.01 (two-tailed); * p < 0.05 (two-tailed).

	Perceived Knowledge score (SKT)	Confidence score (SST)
Years of experience in primary healthcare	0.403**	0.165**
P value	0.001	0.007

## Discussion

This study assessed perceived knowledge, confidence, and practice patterns related to MSPs and IM injections among primary healthcare physicians and compared these findings with existing international literature. Overall, the results demonstrate moderate perceived knowledge, low procedural frequency, and limited confidence, findings that are largely consistent with previous studies conducted in both regional and global primary care settings. Perceived knowledge levels were predominantly moderate, with a considerable proportion of physicians reporting poor or very poor perceived knowledge, particularly in complication management and safe IM injection techniques. Notably, only 52.8% had received formal procedural training, indicating a substantial educational gap. This deficiency likely contributes to the variability in procedural competence observed and suggests insufficient integration of structured surgical skills training within primary healthcare professional development frameworks.

These findings indicate that perceived knowledge alone may not be sufficient to ensure procedural competency in routine clinical practice. Strengthening procedural training during residency and continuing professional development may help bridge the gap between expected competencies and actual clinical performance. These findings are consistent with Collins et al. (2010), who reported inconsistent and insufficient surgical instruction for general practitioners, with wide variation in who is trained, how, and how often [1]. Similarly, Menahem et al. (2014) found that physicians with formal training were significantly more knowledgeable and more likely to perform MSPs [6]. Furthermore, textbook-based references describe MSPs as core primary care competencies, highlighting a mismatch between expected competencies and actual preparedness in real-world primary healthcare settings [3].

A substantial majority of physicians reported rarely or never performing MSPs (71.7%) or IM injections (67.7%), reflecting significant underutilization of procedural capacity within primary healthcare and hospital settings. This pattern closely mirrors findings from Sempowski et al. (2006) [8] and Alfaraj et al. (2015) [7]. In contrast, Menahem et al. (2014) reported higher procedural volumes in settings supported by structured training and institutional facilitation, suggesting that reduced procedural frequency is more likely attributable to systemic and organizational constraints rather than lack of physician interest or scope limitations [6]. The low frequency of performing MSPs and IM injections may reduce the availability of office-based procedural care within primary healthcare, potentially increasing referrals to secondary care for conditions that could otherwise be managed safely in primary care settings.

Physician confidence in performing MSPs was generally low to moderate, particularly for technically demanding procedures such as suturing and abscess drainage. The observed moderate positive correlation between perceived knowledge and confidence (ρ = 0.384) quantitatively supports earlier qualitative observations reported by Maguire (2000) [9] and Menahem et al. (2014) [6]. However, the modest strength of this association suggests that perceived knowledge alone may not fully explain procedural confidence. This finding indicates that procedural confidence is influenced not only by perceived knowledge but also by repeated supervised practice and regular opportunities to perform procedures in routine clinical settings. Given the cross-sectional design of this study, the observed associations should not be interpreted as evidence of a causal relationship.

The most frequently reported barriers were lack of training (68.4%), time constraints (63.6%), and inadequate equipment availability (53.2%). These findings are highly consistent with regional data reported by Alfaraj et al. (2015) [7] and international findings by Sempowski et al. (2006) [8], suggesting that such barriers are systemic rather than context-specific. Additionally, concerns regarding complications align with Lowy et al. (1994), who emphasized that perceived medicolegal and safety risks significantly influence procedural engagement [10]. The convergence of these findings across diverse healthcare systems indicates that organizational infrastructure plays a central role in enabling or constraining minor surgical practice. Because these barriers are largely modifiable, institutional interventions such as protected procedural time, improved resource allocation, and structured training programs may substantially enhance procedural practice in primary healthcare.

Participants strongly endorsed hands-on workshops (77.7%), improved access to procedural equipment, clear clinical guidelines, and mentorship as key facilitators for enhancing procedural practice. These findings align with evidence from Maguire (2000) [9] and Menahem et al. (2014) [6], who demonstrated that structured training programs and organizational support significantly enhance procedural activity in primary care. Primary care procedural frameworks further affirm that MSPs can be safely and effectively performed in well-supported primary care settings. The strong preference for workshops and mentorship highlights physicians’ willingness to expand their procedural skills when adequate educational and organizational support is available.

Years of professional experience demonstrated a stronger association with knowledge than with confidence, suggesting that accumulated clinical exposure may enhance theoretical understanding but does not necessarily translate into procedural self-assurance. This observation aligns with Maguire (2000) [9]. Additionally, the absence of significant gender differences in knowledge or confidence parallels findings by Sempowski et al. (2006) [8], indicating that procedural disparities are more strongly influenced by systemic and educational factors than by demographic characteristics. These findings suggest that improving procedural competency should focus primarily on strengthening educational opportunities and healthcare system support rather than physician demographic characteristics.

In summary, the findings of this study are largely consistent with existing international literature and highlight persistent gaps in training, confidence, and procedural practice among primary healthcare physicians. Addressing these gaps through structured training programs, adequate resource allocation, and institutional support may enhance the integration of MSPs into primary care, reduce unnecessary referrals, and improve patient access to timely procedural care [12].

Strengths and limitations

The strengths of this study include its adequate sample size and the comprehensive assessment of primary healthcare physicians’ perceived knowledge, procedural practice, self-confidence, and perceived barriers related to MSPs and IM injections. The inclusion of physicians from different levels of training and practice settings enhances the representativeness of the findings and provides a broad overview of procedural practice in primary healthcare.

This study has several limitations. First, its cross-sectional design precludes the establishment of causal relationships. Second, the use of convenience sampling and online questionnaire distribution may have introduced selection bias, potentially limiting the generalizability of the findings to all primary healthcare physicians in Saudi Arabia. Third, although the questionnaire underwent expert review for face and content validity by expert family medicine consultants, no formal psychometric validation (e.g., construct validity or factor analysis) was performed. Fourth, perceived knowledge, confidence, and practice were assessed using self-reported questionnaire responses rather than objective assessments of procedural competence, making the findings susceptible to reporting and social desirability bias. Consequently, the reported levels of perceived knowledge, confidence, and practice may not accurately reflect actual procedural competence or the safe performance of MSPs and IM injections.

## Conclusions

This study suggests that PHC physicians have moderate self-reported perceived knowledge but limited self-reported confidence and practice regarding MSPs and IM injections. Many participants reported rarely performing these procedures, with lack of training, limited time, and insufficient equipment identified as the most common perceived barriers. Although greater perceived knowledge was associated with higher confidence, the cross-sectional design of the study does not permit conclusions regarding causal relationships or actual procedural competence.

The findings highlight potential areas for improvement in procedural training and support within PHC settings. Structured hands-on training programs, adequate resource allocation, workflow optimization, and mentorship may help enhance physicians’ confidence and procedural practice. However, further studies using objective assessments of procedural skills are needed to better evaluate clinical competence and patient-related outcomes.

## Appendices

Study questionnaire

You are being invited to participate in a research study titled: “Prevalence, Knowledge, Clinical Skills, Self-Confidence, and Barriers of Primary Healthcare Physicians in Performing Minor Surgical Procedures (MSP) and Intramuscular Injections (IM injections)” in Riyadh, Saudi Arabia. Your participation is voluntary. The survey takes approximately 5 minutes. All responses are confidential and will be used for research purposes only.

1.   By selecting “I Agree” below, you confirm that you are over 18 years of age and consent to participate. 

                          I agree

Section A: Demographics

2. AGE :

3. GENDER

Female

Male

4. Nationality

Saudi

Non-Saudi

5. Highest Qualification

MBBS

Family Medicine Board

Diploma

OTHER: _________________

6. Years of experience in primary healthcare? _________________

7. Current working facility

MOH PHC

MOH Hospital

Other:________________________________

8. Have you received any formal training in minor surgical procedures?

Yes

No

Section B: Knowledge Assessment

( 1= very poor, 2=poor, 3=average , 4-good and5=excellent) Mark only one choice.

9. Your knowledge of aseptic techniques during minor surgical procedures:

☐ Very Poor

☐ Poor

☐ Average

☐ Good

☐ Excellent

10. Your understanding of indications for common minor surgical procedures (e.g., incision & drainage, excision of cysts):

☐ Very Poor

☐ Poor

☐ Average

☐ Good

☐ Excellent

11. Your knowledge of complications associated with minor surgical procedures:

☐ Very Poor

☐ Poor

☐ Average

☐ Good

☐ Excellent

12. Your knowledge of safe sites and techniques for intramuscular injection:

☐ Very Poor

☐ Poor

☐ Average

☐ Good

☐ Excellent

Section C: Skills and Practice

(Answer the following with: (1=Never, 2= rarely , 3= sometimes, 4= often ,5= Always) Mark only one choice.

13. How often do you perform minor surgical procedures in your current practice?

☐ Never

☐ Rarely

☐ Sometimes

☐ Often

☐ Always

14. How often do you perform intramuscular injections?

☐ Never

☐ Rarely

☐ Sometimes

☐ Often

☐ Always

15. How confident are you in suturing superficial wounds? *

☐ Never

☐ Rarely

☐ Sometimes

☐ Often

☐ Always

16.  How confident are you in performing abscess drainage?

☐ Never

☐ Rarely

☐ Sometimes

☐ Often

☐ Always

17.  How confident are you in managing complications post-procedure? *

☐ Never

☐ Rarely

☐ Sometimes

☐ Often

☐ Always

18. Do you use sterilized equipment and standard precautions consistently?

☐ Never

☐ Rarely

☐ Sometimes

☐ Often

☐ Always

Section D: Barriers

(Select all that apply or write your response)

19. What are the main barriers that prevent you from performing minor surgical procedures and IM injections regularly?

☐ Lack of training

☐ Lack of time

☐ Lack of equipment/supplies

☐ Institutional restrictions

☐ Fear of complications

☐ Low patient demand

☐ other ________________________

Section E: Suggestions for Improvement

(Select all that apply or write your response)

20. What would help you perform these procedures more confidently and frequently?

☐ Workshops/Hands-on training

☐ Access to proper instruments

☐ Clear institutional guidelines

☐ Supervisory support/mentorship

☐ other: _________________________

21. Do you have any additional suggestions to improve the practice of minor procedures and IM injections in PHC settings?

       ______________________________________________________________________________________
